# A Mobility Aware Binary Tree Algorithm to Resolve RFID Jam and Bottleneck Problems in a Next Generation Specimen Management System

**DOI:** 10.3390/mi11080755

**Published:** 2020-08-04

**Authors:** Yen-Hung Chen, Yen-An Chen, Shu-Rong Huang

**Affiliations:** 1 Department of Information Management, National Taipei University of Nursing and Health Sciences, Taipei 112, Taiwan; 2 Taipei Veterans General Hospital, Taipei 112, Taiwan; 3 Department of Information Management, National Chiao Tung University, Hsinchu 300, Taiwan; hsr.long@gmail.com

**Keywords:** RFID, tag identification, anti-collision, mobile tag, specimen management

## Abstract

Hospitals are continuously working to reduce delayed analysis and specimen errors during transfers from testing stations to clinical laboratories. Radio-frequency identification (RFID) tags, which provide automated specimen labeling and tracking, have been proposed as a solution to specimen management that reduces human resource costs and analytic delays. Conventional RFID solutions, however, confront the problem of traffic jams and bottlenecks on the conveyor belts that connect testing stations with clinical laboratories. This mainly results from methods which assume that the arrival rate of specimens to laboratory RFID readers is fixed/stable, which is unsuitable and impractical in the real world. Previous RFID algorithms have attempted to minimize the time required for tag identification without taking the dynamic arrival rates of specimens into account. Therefore, we propose a novel RFID anti-collision algorithm called the Mobility Aware Binary Tree Algorithm (MABT), which can be used to improve the identification of dynamic tags within the reader’s coverage area and limited dwell time.

## 1. Introduction

Currently, many medical institutions have begun using RFID [1,2,3] for the internal management of drug administration, blood transfusions, patient identification, and surgical equipment, as well as the collection of surgical information. Radio frequency identification is widely used in automated identification systems. It is more convenient and immediate than traditional barcode systems, and there is no need to handle the object to be identified. An RFID system includes a reader and several tags, each with a unique identifier (ID), which allows the reader to identify all tags within its coverage area via radio signals. However, collision, which means different tags send signals to the reader simultaneously, may occur during the tag identification process. Collision causes that the reader cannot immediately identify any tags that cause collisions, resulting in a waste of bandwidth resources and prolonged tag identification time. In the medical field, accuracy and efficiency are vital. Therefore, designing a good anti-collision algorithm for RFID tag identification is necessary.

Several RFID anti-collision algorithms have been proposed to improve the speed of tag identification and minimize delays in the tag identification process. These RFID anti-collision algorithms can be broadly categorized as Aloha-based [4,5] or Tree-based [6,7]. Aloha-based algorithms set up an appropriate number of slots in a frame, which determines the frame length. During the recognition process, these algorithms then work to minimize the frame length by observing the ratio of collisions to successes in each frame. Tree-based algorithms, on the other hand, continuously divide the colliding tags into two subgroups until each group has only one or no tags. Tree-based algorithms can be further divided into Query tree (QT) algorithms and Binary tree (BT) algorithms. Collision tags for QT algorithms are grouped according to the query conditions transmitted by the reader, while in BT algorithms they are randomly selected as a binary value for grouping.

However, in the actual RFID environment, these traditional anti-collision algorithms do not consider that tags continuously move in and out of the reader’s coverage area and dwell time within the coverage area is limited, so they do not give priority to tags that are about to leave the coverage area. Moreover, hospitals need to examine a huge number of specimens, and tags may be placed on anything from specimens on a conveyor belt to packages of prescribed drugs for automatic sorting [8,9,10,11,12]. It is important to consider not only the length of time during which tags stay in the reader’s coverage area, but also a mechanism to prioritize the tags that are about to leave it.

A schedule-based anti-collision protocol (SAC) [13] solves the problem of identifying dynamic tags moving in and out of the coverage area in the traditional algorithms described above. SAC uses two readers to identify dynamic tags. The first reader assigns a group ID to each tag, while the second identifies the tags and prioritizes those that are about to leave the coverage area based on their group ID. However, SAC has two disadvantages. The first is that it uses a fixed frame length. When the tags move faster, the number of tags that pass by is too great for the fixed frame length to cope with. This results in a large number of collisions and a severely reduced recognition rate. Second, the starvation problem of Aloha-based algorithms, in which tags are continuously colliding and cannot be identified, also exists in SAC.

The goal of this study was to develop a novel RFID anti-collision algorithm to improve the identification of dynamic tags. This algorithm, called the Mobility Aware Binary Tree Algorithm (MABT), can be used to improve the identification of the dynamic tags within a reader’s coverage area and limited dwell time. The MABT anti-collision algorithm has three features: (1) grouping to prioritize tags that are about to leave the coverage area of the reader; (2) estimation of the number of tags and setting of an appropriate number of time slots per frame (to reduce collisions due to different tags choosing the same time slot, effectively use idle time slots, and increase the accuracy of recognition); and (3) switching to BT identification when tags collide due to random selection, which can reduce recognition time delays and effectively avoid hunger. The introduction of this novel anti-collision RFID algorithm in the medical industry can result in greater precision, less manpower, and fewer errors in specimen management, thus improving efficiency and patient safety.

## 2. Background

### 2.1. Overview of RFID System

**Figure 1:**
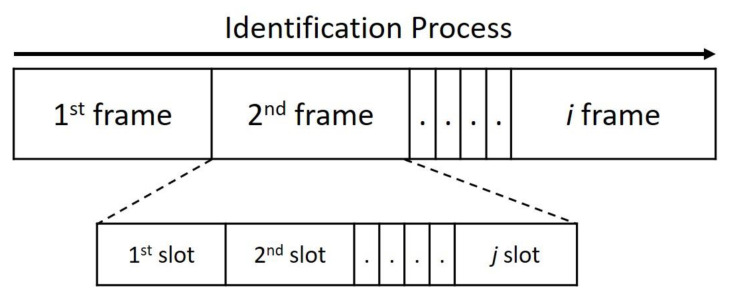
Relationship between frame and time slot.

**Figure 2:**
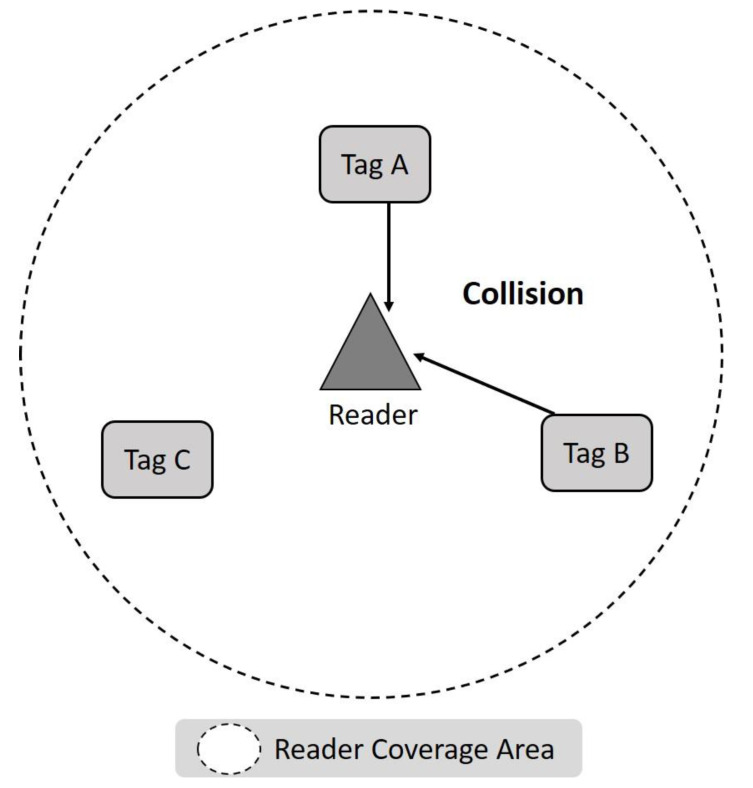
Collisions in a radio-frequency identification (RFID) system.

Typically, existing RFID systems consist of a single reader and multiple tags, and they automatically identify or track objects with the embedded or attached tags. Figure 1 shows some of the technical terms used in the identification process. The frame is also called a recognition cycle and refers to the time from which a reader sends a signal until it receives and processes a tag ID within its coverage area. Therefore, the recognition process is divided into many frames. We define *f_i_* as the *i* frame, which contains a set of slots. Each slot represents a kind of feedback the reader should send to the tag based on the result of the received signal. We define *S_i, j_* as the *j* time slot in the *i* frame. It should be noted that the status of each time slot can be classified into three types: Idle, Success, and Collision. When the slot is Idle, there is no tag to respond to, and the reader will not receive any signal. Success means that the reader can successfully decode the signal with the ID of one or more tags. If the reader receives a signal but cannot decode any information from it, it is called a Collision slot. As shown in Figure 2, a Collision is usually due to the slot sending a signal to the reader when two or more tags choose the same slot.

During the identification process, the reader broadcasts periodically (Broadcast), which means it sends a query or message to each frame. If a tag within the reader’s identification coverage area meets the query conditions, it will choose a time slot in which to respond to the reader with its ID. Ideally, to maximize bandwidth usage, the number of time slots in a frame should be equal to the number of tags within the reader’s coverage area. If the frame length is greater than the actual number of labels, there may be time slots that are not effectively used; if the frame length is less than the actual number of labels, the labels may suffer severe collisions. Therefore, the RFID reader must estimate changes in the number of tags in order to properly determine frame length.

### 2.2. Dynamic RFID System Model

**Figure 3:**
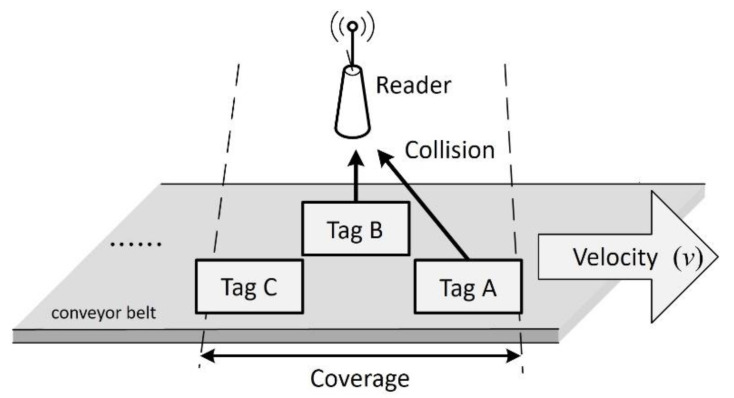
Dynamic RFID system.

A dynamic RFID system has a fixed RFID reader and several dynamic RFID tags which enter and exit the coverage of the reader. Once commonly used in warehouses and stores for automatic sorting and distribution, these systems are now also used in the medical field for specimen conveyor belt management [8,9,10,11,12]. As shown in Figure 3, a conveyor belt moves the tags at a constant transport rate of *v*. The reader is placed over the conveyor belt to detect the tags, which enter and leave the reader’s fixed coverage area from left to right. Figure 3 shows two tags labeled Tag A and Tag B entering the reader’s identification range at different times. If the two Tags A and B keep colliding during the identification process, the reader’s inability to know which tag will leave the coverage area first makes it impossible for the reader to identify them successfully within a limited time.

**Figure 4:**
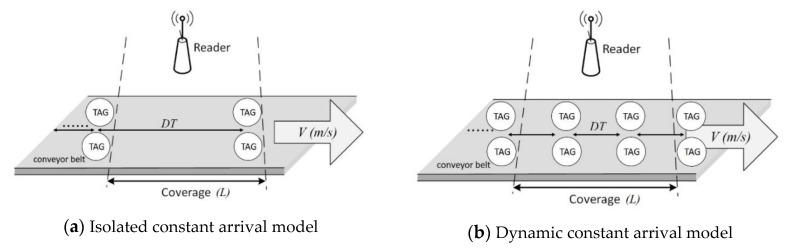
Examples of dynamic tag models.

Models for dynamic tags can be classified according to two factors: the number of tags in the interrogation zone (NT) and the distance between consecutive tags (DT). If NT maintains a constant value at any time, it is called constant arrival, as shown in Figure 4. If NT changes over time, it is called variable arrival. When DT is greater than or equal to the coverage area of reader (*L*), which means that only one tag will be in coverage area at any given time, it is called isolated arrival. Otherwise, it is called dynamic arrival.

Using different combinations of the factors NT and DT, four basic types of dynamic tag can be formed: isolated constant arrival, isolated variable arrival, dynamic constant arrival, and dynamic variable arrival. It should be noted that constant arrival and dynamic arrival do not conflict, because the number of tags within the coverage area can be kept at a constant value by continuously changing the number of tags in and out per time unit.

### 2.3. Discussion in Related Literature

RFID anti-collision algorithms are mainly Aloha-based [14,15] or Tree-based. In Aloha-based algorithms, the recognition process is divided into many frames and an appropriate number of time slots are included in each frame, which determines the length of the frame. The reader adjusts the number of time slots based on the collision, success, and idle time of each time slot in the frame in an attempt to minimize the frame length during the recognition process. Q algorithms and Dynamic Frame Slotted Aloha (DFSA) algorithms are used to generate appropriate frame lengths. The former uses the recognition result of each time slot in a frame to adjust the Q value (an integer) to restart a new frame with a length of 2Q for random selection of unidentified labels. The latter directly sets the frame length by estimating the number of tags and giving the appropriate number of time slots. One method of estimation is the Low-bound method. A collision consists of at least two tags responding at the same time, so *EstLow-bound* = *number of collisions* × 2. Although Aloha-based algorithms are more suitable for dynamic environments, they may lead to potential hunger problems.

Tree-based algorithms continuously divide tags that have collided into two sub-groups until each group has only one label or no labels exist. They can be further divided into QT algorithms [16,17] and BT algorithms [18,19,20].

In QT algorithms, the reader first generates two queries, “0” and “1”, to the tags in the queue. Then, these tags respond after comparing the prefix from their ID with the query *q* from the reader. When a collision occurs in a slot, the reader extends two new queries, *q*+ “0”, *q*+ “1” into the queue until all the tags are successfully identified when it becomes empty. In BT algorithms, there is a counter for each tag and reader. The counter in the tag records the time slot when the tag has to wait. When the tag’s counter zeroes, it sends its ID to the reader, and the reader’s counter records the time slot that is waiting for the reader. Therefore, when the reader finishes recognition of the last slot, the counter goes to −1, which means that all time slots have been processed. At this point, the grabber terminates the frame. With tree-based algorithms, it may not be easy to estimate the number of unrecognized tags in recognition under a dynamic situation, but, because of its short recognition delays, BT algorithms are widely used in large, complex RFID systems.

However, traditional anti-collision algorithms were designed primarily for use in static RFID systems and not for dynamic tags entering and leaving the reader’s coverage area. If a tag enters the reader in frame *f_i_*, it will leave after frame *f_i_ + n* whether the tag has been successfully identified or not, which also indicates that the tag’s residence time in the coverage area is limited. The successful identification rate will also dramatically decrease, once the arrival rate of the incoming tags is dynamic (called a dynamic RFID system). This is because the conventional RFID anti-collision algorithms do not distinguish the incoming order and movement direction of continuous incoming tags. Since the reader cannot prioritize tags that are leaving the coverage area, the rate of successful identifications will decrease as the conveyor belt moves faster. Therefore, RFID readers must be able to give priority to tags that are about to leave coverage considering the continuous movement of tags and the relationship between tags in a dynamic tag model, when these readers are set up to read continuously and immediately find increments of incoming RFID tags.

**Figure 5:**
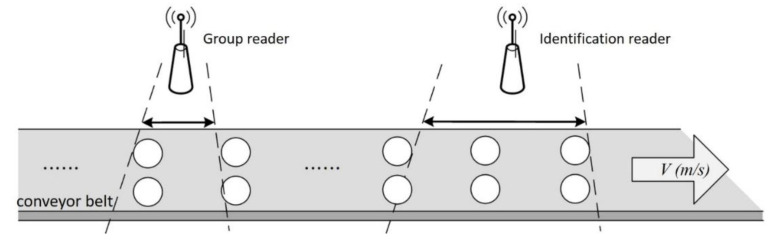
Schedule-based anti-collision protocol (SAC) algorithm setup.

SACs [13] solve the problem of limited time for identifying dynamic tags in the coverage area. SAC algorithms use two readers to identify dynamic tags, as shown in Figure 5. The first is the group reader, which assigns a group ID to the tags that have entered its coverage area; the smaller a group ID is, the earlier that group has entered the coverage area. The second is an identification reader. The mechanism for identifying tags in the coverage area is based on the group ID. Therefore, the smaller is the group ID, the sooner it will leave the coverage area, and the higher its priority in processing identification. However, SAC has two disadvantages. The first is that it uses a fixed frame length. Because the number of labels is not estimated, it is easy to overestimate or underestimate the actual required frame size. As labels move more quickly, the fixed frame length cannot handle the large number of labels entering and leaving, which leads to an increase in the number of idle and collision slots and a serious decrease in the recognition rate. The second problem is that SAC algorithms still have the starvation problem of Aloha-based algorithms. Even if the group ID can be processed in order, there are still cases that cannot be identified due to tags continuously colliding.

## 3. Materials and Methods

This study proposes a motion-aware binary tree algorithm (MABT) to improve the identification of dynamic tags with limited dwell time in a reader’s coverage area. The goal is to achieve an acceptable tag recognition rate in an environment of high-density tag quantities and high-speed tag movement by combining the advantages Aloha-based and Tree-based anti-collision algorithms. Aloha-based algorithms are suitable for a dynamic environment by randomly select time slots within a given frame length during the recognition process. Tree-based algorithms are characterized with specific query conditions sent by the reader, which can shorten recognition delays during the recognition process and ensure that hunger does not occur.

In Section 3.1, we explain how to determine optimal frame length, then describe the operational flow of the MABT algorithm in Section 3.2, and give an example of the MABT algorithm in Section 3.3.

### 3.1. Determining Optimal Frame Length

With dynamic tags, an RFID anti-collision algorithm needs to achieve two goals: workload optimization and identification deadline prioritization. Workload is defined as the number of tags competing for the same time slot at the same time; identification deadline is defined as the time point before each tag leaves the coverage area. The priority of workload optimization is to maintain a stable quantity of accurate identifications, which means the identification rate should be greater than the moving speed of the conveyor belt. The identification deadline prioritization ensures that the tag can be detected early when it enters the end of the reader’s coverage area, which means that tags at the back of the coverage area will have higher recognition priority than those in the front or newly entered tags.

To optimize the workload, it is necessary to estimate the number of tags to be processed in each frame. Both the number of unrecognized tags and the number of newly entered tags must be estimated. Precisely estimating the total number of tags and giving appropriate time slots helps avoid the unnecessary waste of time slots or excessive collisions, but the complexity of estimation may lead to longer recognition delay times or missed opportunities to identify labels. Therefore, it is important to calculate the number of frames and slots for tags to randomly select based on successfully identified tags, unrecognized tags due to collision, and expected increase of new tags.

According to RFID time series and historical data in dynamic RFID environments, if the time series can reasonably be postponed, then past data can be used to predict the future. Although unrecognized tags can still be estimated in traditional algorithms, they cannot be directly used to determine frame length because the extra tags (or tag groups) must be considered. Therefore, this paper proposes to use the exponential smoothing method to estimate the number of labels to determine optimal frame length; that is, to use the weighted average of the past time series to smooth the data, and use the smoothed weighted average as the predicted value for the next period. The basic formula for this exponential smoothing method is shown in Equation (1):

(1) $$F_{t+1}=\alpha \times X_{t}+\left(1-\alpha \right)\times F_{t}$$

*F_t_* is the predicted value in period *t*; *F_t_* + 1 is the predicted value in period *t* + 1; *X_t_* is the actual demand value in period *t*; and *α* is the smoothing constant, i.e., the error correction coefficient. As a sensitivity adjustment to the prediction error, its value must be between 0 and 1. If *α* is closer to 0, past observations will be weighted more heavily. By contrast, when *α* is closer to 1, more recent observations are weighted. The exponential smoothing method has two additional attributes: distance from the forecast period and number of observed data sets. This is because a larger weight should be given to observation values closer to the forecast period or larger numbers of observed datasets. The purpose here is not to abandon past observations, but to give them a gradually weakening degree of influence.

Finally, based on the attributes described above and the method for estimating unidentified labels proposed by Cha and Kim [4,18], a new equation (Equation (2)) for dynamic environments can be derived in which *N_succ_* represents each superframe and *F_Ncoll_* represents the number of time slots for each collision within a frame.

(2) $$F_{t+1}=\alpha \times \left(FN_{coll}\times 2.3922+N_{succ}\right)+\left(1-\alpha \right)\times F_{t}$$

Identification deadline prioritization refers to how to allocate the best group size. The faster is the speed, the more tags will enter the coverage area, resulting in a severely increased rate of collisions. To effectively solve this problem, it is necessary to limit the number of tags so that they can respond in the frame to reduce collisions. This concept is called pre-grouping. In addition to being able to achieve a low rate of collisions by limiting the tags that can respond in the frame, an optimal group size also gives tags that are about to leave the reader’s coverage area a higher recognition priority than tags that have just entered. By estimating the average number of tags in each time slot that the reader is broadcasting to, the time interval of each broadcast can be adjusted using the exponential smoothing method, so that each group can be made up of the most suitable number of labels.

Newly added tags and unrecognized tags need to be counted for estimation, and *α* will adopt a specific value because it is in a stable state over the long-term.

### 3.2. MABT Algorithm

**Figure 6:**
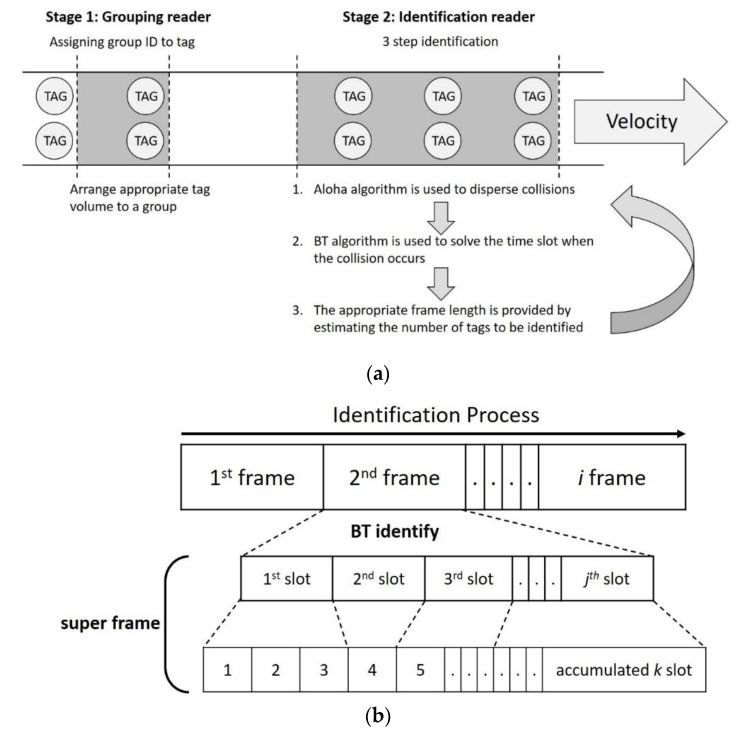
Concept of mobility aware binary tree algorithm (MABT): (**a**) MABT identification algorithm design; and (**b**) relationship between super frame and time slot.

To achieve the above two goals, the MABT algorithm uses two RFID readers and two stages of identification processing, as shown in Figure 6. The setup is shown in Figure 6a. The grouping reader is on the left, while the identification reader is on the right. The two readers are both equipped with single antenna. It should be noted that the grouping reader is placed before the identification reader, which means the tags will not be recognized until they are grouped. Furthermore, the distance between two RFID readers and the corresponding conveyor belt should be adjusted based on the triangulation and strength of radio signal regarding the tag identification environment.

The MABT algorithm is divided into two stages. The first stage is assigning a group ID to a tag, and the second stage is identifying the tag. The purpose of the first stage is to arrange the most appropriate quantity of tags to the same group ID through the group reader, so that the number of tags in each group is as similar as possible. The purpose of the second stage is to effectively use the time slots and decrease the probability of collisions between labels. Finally, the two stages are cycled to continuously identify the tags. (1) The appropriate frame length is provided by estimating the number of tags to be identified. (2) A BT algorithm is used to resolve time slots when collisions occur. (3) When the accumulated number of time slots reaches the default interruption standard, it will stop recognizing the current frame and initialize recognition of the next frame. The default interruption standard mentioned above refers to the length of the super frame, which is the accumulated time slots used, as shown in Figure 6b. During the identification process, each frame has *j* time slots. In the BT method, when time slot collisions occur, two new time slots are generated, after which identification continues in the new time slot. Conversely, if a time slot is successfully identified or idle, no new time slot will be generated. When a new time slot is generated, the number of time slots used also accumulates; thus, when the preset super frame length is reached, the identification of this frame will end.

The receiveGroupingReader function is executed at each time slot, where the frame length *f* indicates that there are *f* time slots in the frame, and that time slot *s* is coded from 1 to *f*.

**Figure 7:**
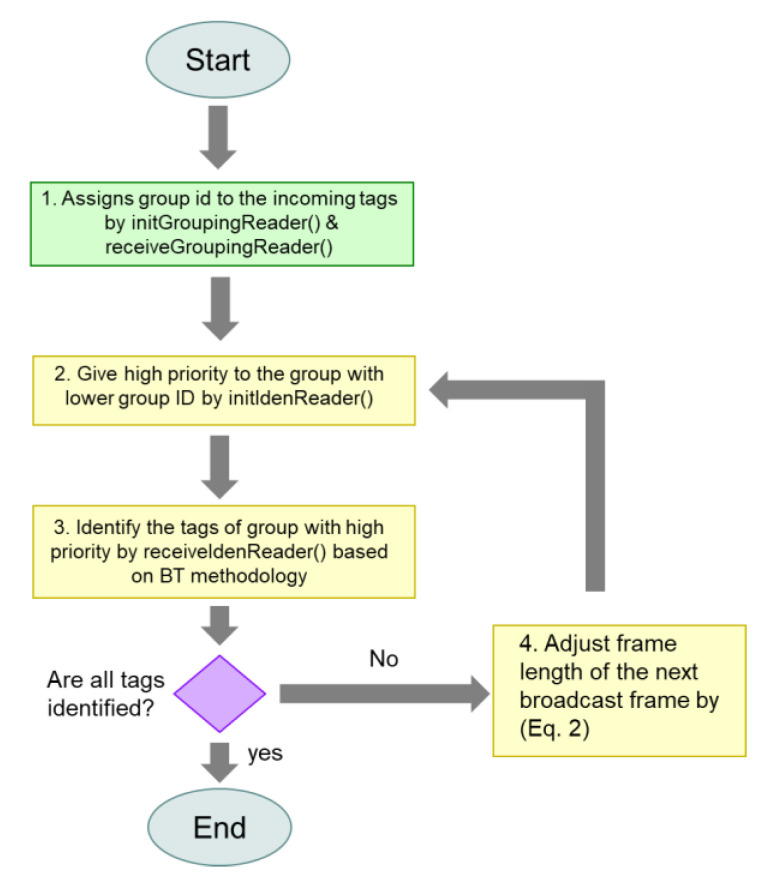
Flowchart of MABT.

**Figure 8:**
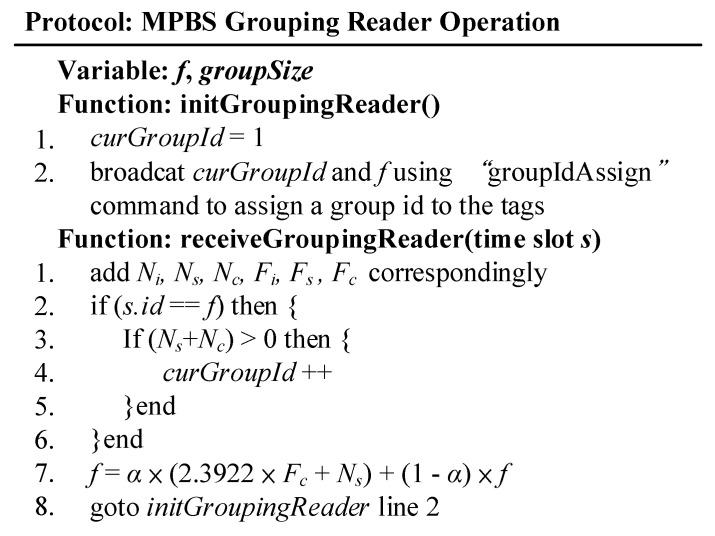
Code for group reader.

**Figure 9:**
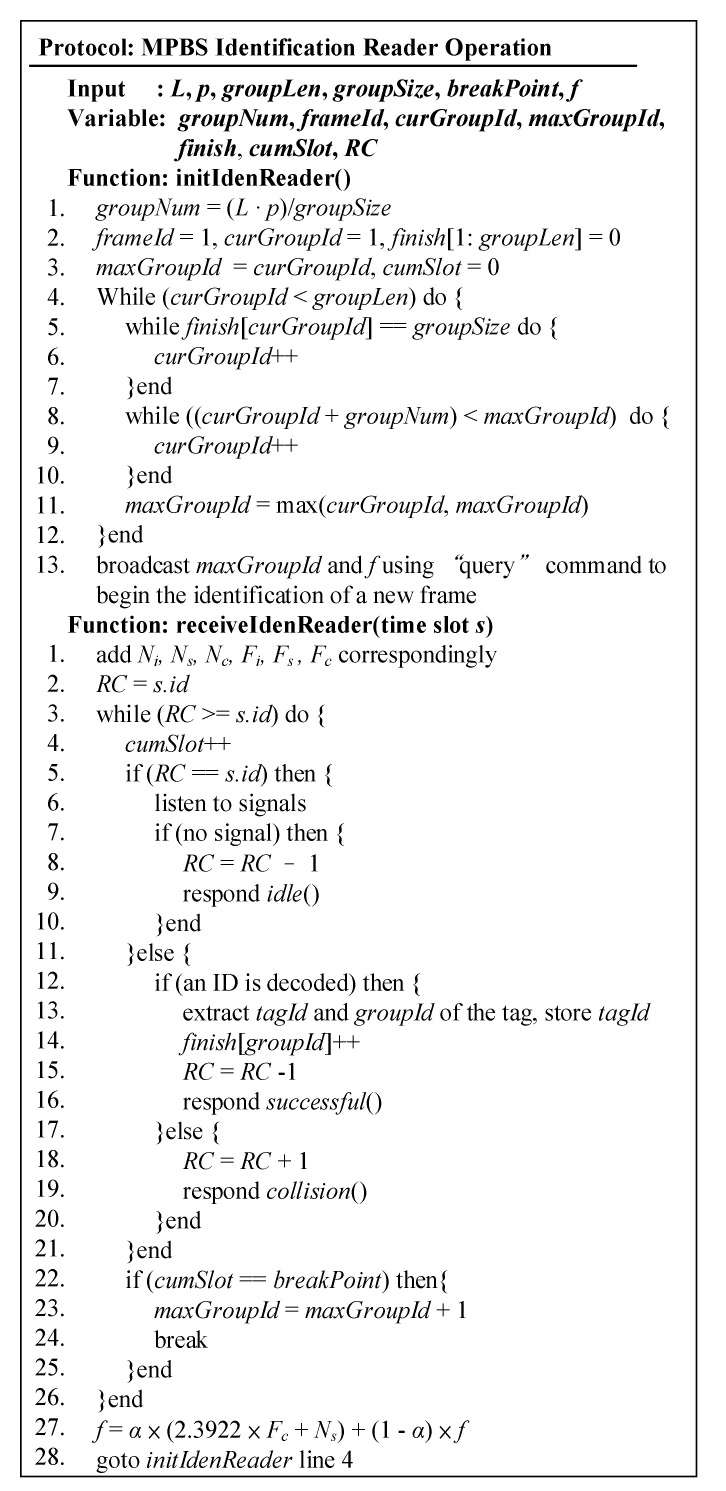
Code for identification reader.

**Figure 10:**
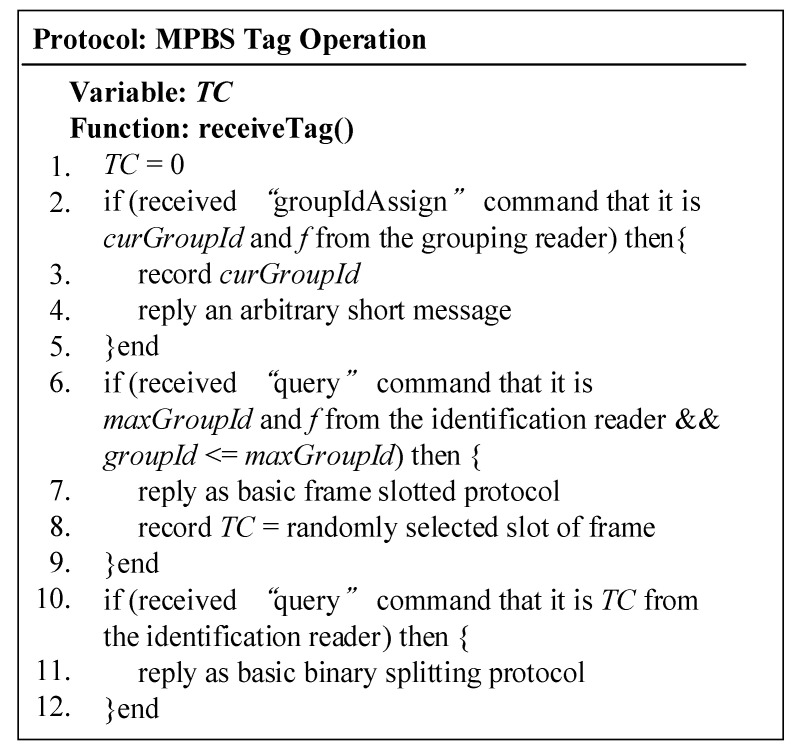
Tag code operation.

The flowchart of MABT is shown in Figure 7, and the detailed operations among the grouping reader, identification reader, and tag are explained in Figure 8, Figure 9 and Figure 10. The readers are capable of initializing and receiving messages. The symbols for and definitions of related parameters are described in Table 1.

The packet reader agreement (Figure 8) is the first stage in Figure 6. It is responsible for assigning a group ID to the tag. In the initGroupingReader function, curGroupId and f are broadcast to the tag by defining a “groupIdAssign” command equivalent to the query condition. After receiving the command, the tag signals back a message which is used as a basis for determining whether the tag is within the coverage area of the reader, and also as a reference for adjusting the frame length. When the judgment result of *s.id* == *f* on Line 2 is true, (*N_s_* + *N_c_*) > 0 is further used to determine whether there is a label in the coverage area. When the result is true to indicate that there is a label, a *curGroupId* value is added; if there is no label, the *curGroupId* value is not changed. Line 7 adjusts the frame length of the next broadcast based on *N_i_*, *N_s_*, *N_c_*, *F_i_*, *F_s_*, and *F_c_* in each frame to suit the current belt speed.

Figure 9 is the identification reader protocol. When identifying tags, it uses the tracking group ID to determine which tags to prioritize. *L*, *p*, and *breakPoint* are defined as input parameters in the system model; *f* and *breakPoint* are the default values; *groupLen* is the total number of groups on the conveyor belt. If this value is unknown, the user can simply set a large enough value. The *groupSize* is the number of tags in a group. If the conveyor speed is known in advance, the number of tags can be calculated. Otherwise, the optimal group size in SAC [13] is used.

In the initIdenReader function, Lines 1–3 initialize the decision variable which resets *cumSlot* to zero for subsequent usage of the time slot. Lines 4–12 refresh the *curGroupId* and *maxGroupId* at the beginning of each new frame as a baseline for tracking to control workloads. Lines 5–7 add one group after the next group has been identified, and Lines 8–12 exclude groups that have left the coverage area of the identification reader. Line 11 obtains the largest identifiable group ID value based on the above results. Finally, on Line 13, the identification reader sends a “query” command containing *curGroupId* and f to the tags.

The receiveIdenReader function uses the BT algorithm to identify each time slot, and *cumSlot* for accumulated time slot usage as the basis for interruption of identification. On Line 1, *N_i_*, *N_s_* and *N_c_* are the cumulative number of idle, successful, and collisions in time slot *k*. *F_i_*, *F_s_* and *F_c_* are the number of idle, successful, and collisions in the first layer of the frame, which is the status of *j.* Lines 2–26 identify the conditions in each time slot using the BT algorithm. Line 2 makes *RC* equal to the current time slot code. Lines 22–25 observe time slot usage to control the identification time limit. Thus, when the cumulative number of slots (*cumSlot*) meets the preset breakpoint (breakpoint), the current identification of this group is ended. The rest of the tags participates in the identification of the next frame together with next group and increases the value of *maxGroupId*. Line 27 estimates the length of the next frame according to *N_i_*, *N_s_*, *N_c_*, *F_i_*, *F_s_*, and *F_c_*. Line 28 returns to the initIdenReader function to start a new round of frames.

Figure 10 shows the operational details of the label agreement. Each label has a TC with an initial value of 0. On Lines 2–5, the tag receives the “query” command from the grouping reader, records the *curGroupId* as the *groupId*, and then returns a short message. On Lines 6–9, when a “query” from the reader is received, it checks whether the *groupId* of the tag is no more than the *maxGroupId* command. If the condition is satisfied, the tag will randomly select a time slot using the Aloha-based algorithm and set the value of TC to the selected time slot. On Lines 10–12, when the tag receives the “query” from the identification reader asking for the TC condition, it responds with the BT algorithm.

### 3.3. MABT Example

**Figure 11:**
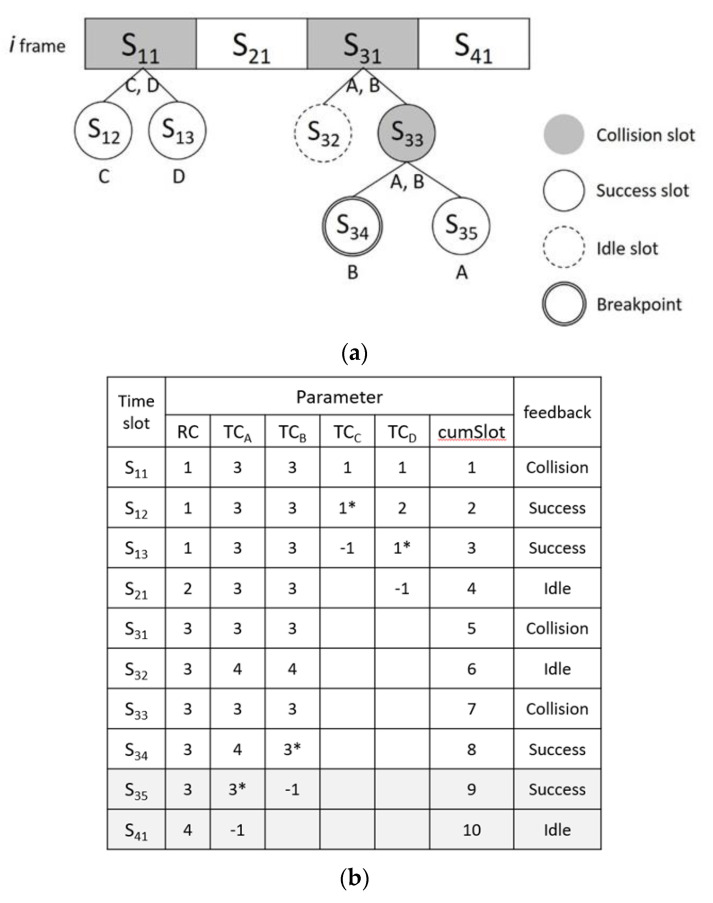

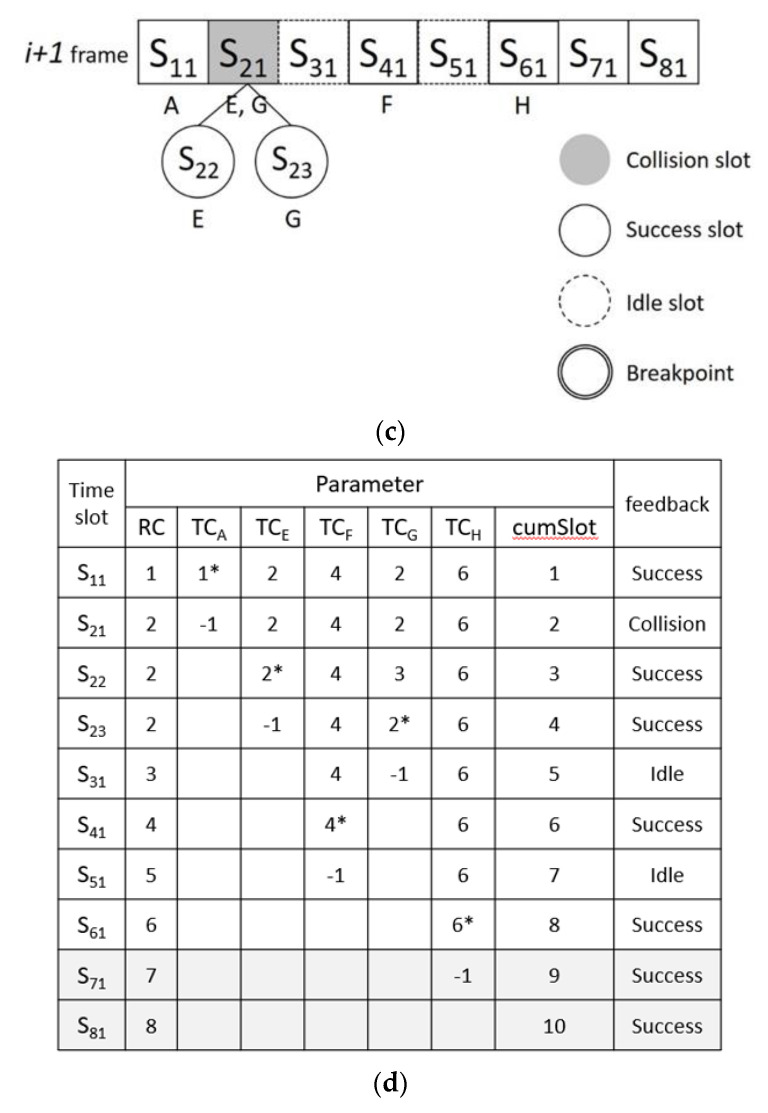
MABT example: (**a**) frame *i*; (**b**) parameters of frame *i*; (**c**) frame *i* + 1; and (**d**) parameters of frame *i* + 1.

In this section, an example is used to illustrate the MABT algorithm. Figure 11 is an example of MABT. In frame *i*, it is assumed that there are four tags—labeled *A*, *B*, *C*, and *D*—for Group 1. In frame *i* + 1, four tags for Group 2—*E*, *F*, *G* and *H*—are added. Figure 11a–d shows information related to the identification tags. A tag randomly selects a time slot in the frame and sets the *TC* value to that time slot. The reader sequentially identifies each time slot and sets the *RC* value of the query condition as the time slot during judgment. The judgment method is based on the BT algorithm. The difference is that the query condition is based on the time slot instead of 0. When a tag has been successfully identified, it is set to −1 because it will be quiet and no longer participate in recognition. The parameter *cumSlot* is used not only to calculate how many time slots are to be used in this frame, but also as the basis for the break point. Suppose that the frame will be interrupted after eight time slots. Even if, as in in Figure 11b, tag *A* can be successfully identified at the 10th time slot, the reader will be forced to end the identification of this frame at the eighth time slot. The purpose of this mechanism to limit identification time is to set a breakpoint to avoid time delays caused by repeated collisions. However, the unrecognized tags in frame i will continue the recognition process in frame *i* + 1 and can be in the same frame with different groups at the same time. There are two points worth noting. First, regardless of the TC value of the tag, a time slot must be randomly selected at the beginning of each new frame. In Figure 11b,d, TC = 3 for label A in frame *i*, but TC = 1 in frame *i* + 1. Second, the frame length is adjusted after frame recognition is completed or interrupted, which brings the number of time slots in the frame closer to the number of tags to be identified and reduces the chance of collision. Thus, in the example, the following information can be obtained in the *i* frame: the frame length *F_t_* = 4, the time slot where the original frame experienced collision *FN_coll_* = 2, the number of successfully identified labels *N_succ_* = 3, and *a* = Case of 0.6. Thus, frame *i* + 1 becomes 8 according to Equation (2).

## 4. Results

We conducted a simulation to evaluate the performance of the MABT and SAC algorithms and compared recognition rates based on the movement speeds of different conveyor belts and tag cooperation. Recognition rate refers to how many tags have been successfully identified out of the total number of tags. The movement rate equals one unit of movement per second. The combination of tags can consider three measurement indicators: average distance, average density, and recognition breakpoint. The purpose of including these three factors was to evaluate: (a) whether the tags could be read within the time limit; (b) the results when there are a large number of tags within the time limit; and (c) the pros and cons of using the time slot and the tag group.

**Figure 12:**
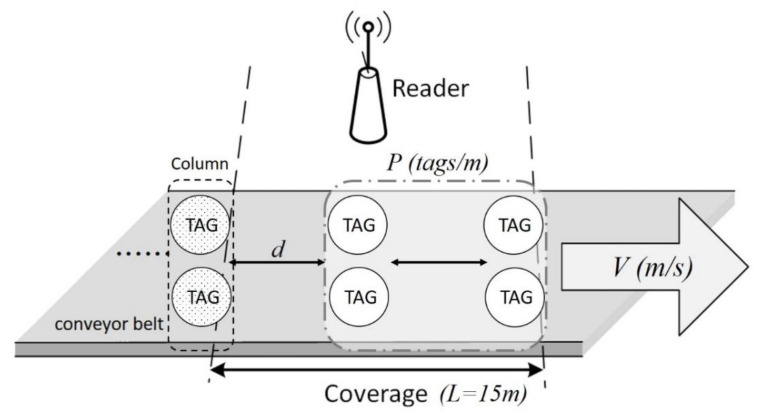
Simulated environment.

To ensure a fair comparison of the MABT and SAC algorithms, the simulated environment settings used were identical, as shown in Figure 12. The environmental parameters of the system model are shown in Table 2. The preset values were set to *L* = 15 m, *d* = 50 cm, and *T_0_* = 15 ms. Other values were adjusted according to different simulation purposes. In addition, the MABT algorithm estimates optimal frame length by estimating the number of labels. Therefore, the initial frame size setting was a fixed frame size using the SAC algorithm as the initial value. Since MABT and SAC use the same estimation formula to determine optimal group size, the same value can be used.

The simulator adopted in this study is extended by simulator developed by our previous works [21,22,23,24]. To ensure the experiments were simulated in a verisimilar environment, we derived the corresponding numerical analysis model in Ref. [21,22,23,24] by the following equations.

The total number of slots consumed by BT to identify n tags, denoted by *S_BT_*(*n*), can be represented as

(3) $$S_{\mathrm{BT}}\left(n\right)=R_{\mathrm{BT}}\left(n\right)+C_{\mathrm{BT}}\left(n\right)+I_{\mathrm{BT}}\left(n\right)$$

$$\begin{array}{c} =\sum_{i=0}^{n}\left(\begin{array}{c} n\\ i \end{array}\right)2^{-n}\left[1+S_{\mathrm{BT}}\left(i\right)+S_{\mathrm{BT}}\left(i-1\right)\right]\\ =\frac{1+2\cdot 2^{-n}\sum_{i=0}^{n-1}\left(\begin{array}{c} n\\ i \end{array}\right)S_{\mathrm{BT}}\left(i\right)}{1-2\cdot 2^{-n}},\;\;\left\{\begin{array}{c} S_{\mathrm{BT}}\left(0\right)=1\\ S_{\mathrm{BT}}\left(1\right)=1 \end{array}\right\} \end{array}$$

where *R_BT_*(*n*), *C_BT_*(*n*), and *I_BT_*(*n*) are the numbers of readable slots, collision slots, and idle slots, respectively. *R_BT_*(*n*), *C_BT_*(*n*), and *I_BT_*(*n*) can be derived as

(4) $$R_{BT}\left(n\right)=n$$

(5) $$\begin{array}{cc} C_{BT}\left(n\right) & =\sum_{i=0}^{n}\left(\begin{array}{c} n\\ i \end{array}\right)2^{-n}\left[1+C_{BT}\left(i\right)+C_{BT}\left(i-1\right)\right]\\ & =\frac{1+2\cdot 2^{\cdot n}\sum_{i=0}^{n-1}\left(\begin{array}{c} n\\ i \end{array}\right)C_{BT}\left(i\right)}{1-2\cdot 2^{-n}},\;\;\left\{\begin{array}{c} C_{BT}\left(0\right)=0\\ C_{BT}\left(1\right)=0 \end{array}\right\} \end{array}$$

(6) $$\begin{array}{cc} I_{BT}\left(n\right) & =\sum_{i=0}^{n}\left(\begin{array}{c} n\\ i \end{array}\right)2^{-n}\left[I_{BT}\left(i\right)+I_{BT}\left(n-i\right)\right]\\ & =\frac{2\cdot 2^{-n}\sum_{i=0}^{n-1}\left(\begin{array}{c} n\\ i \end{array}\right)I_{BT}\left(i\right)}{1-2\cdot 2^{-n}},\;\;\left\{\begin{array}{c} I_{BT}\left(0\right)=1\\ I_{BT}\left(1\right)=0 \end{array}\right\} \end{array}$$

Our designed simulator was validated by Equations (3)–(6) in our previous works [21,22,23,24], which demonstrated the mathematical analysis results match the simulation results of our designed RFID simulator very well.

**Figure 13:**
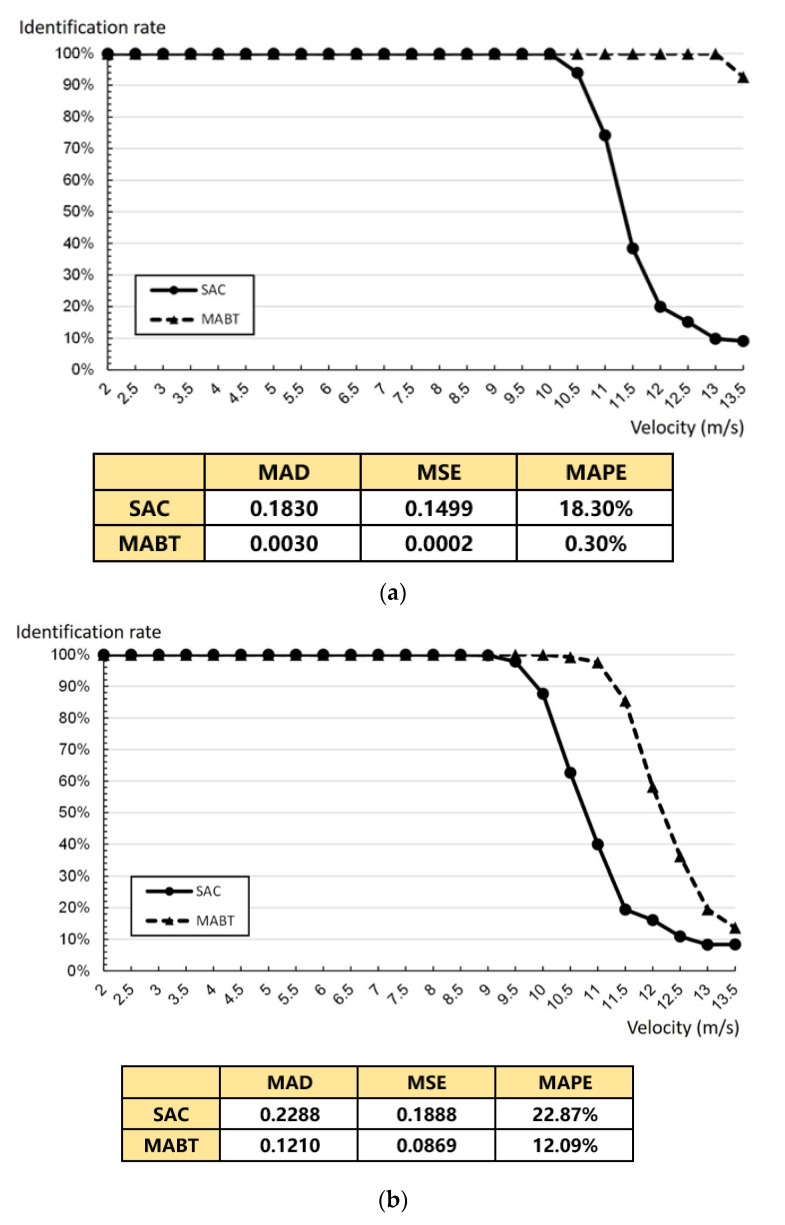

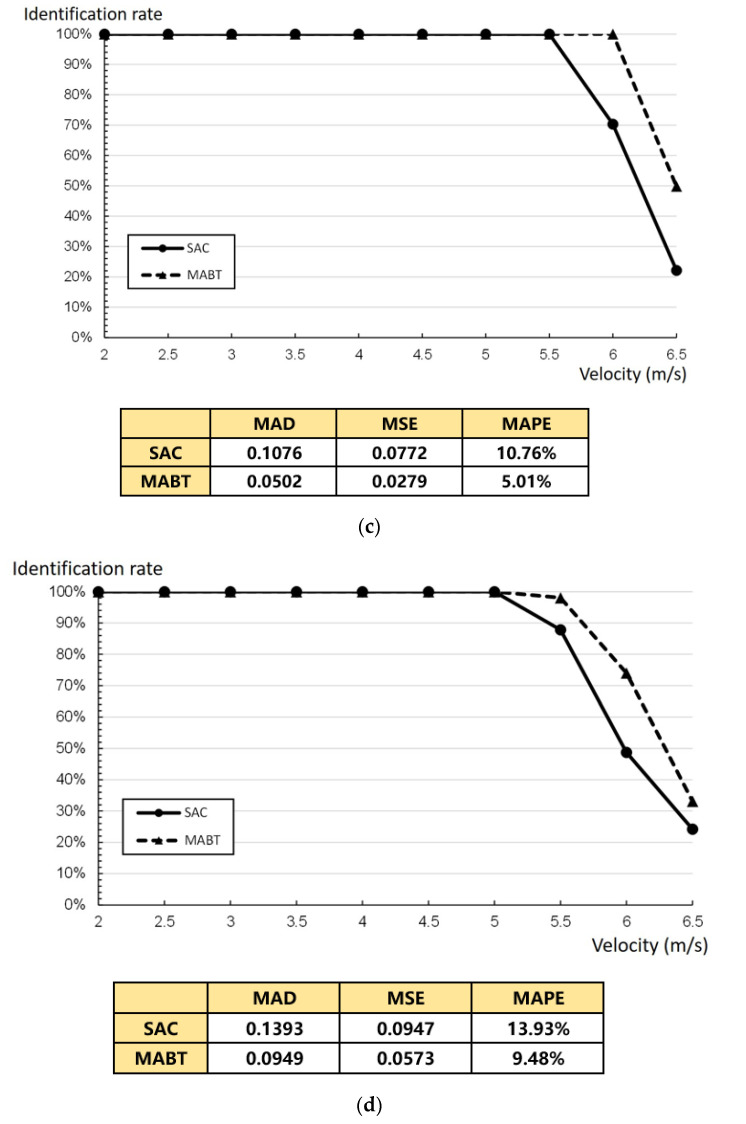
Average distance between tags: (**a**) fixed distance with one tag/column; (**b**) variable distance with one tag/column; (**c**) fixed distance with two tags/column; and (**d**) variable distance with two tags/column.

As shown in Figure 13, the rate of the conveyor belt was one meter per second (1 m/s), and measurements were in units of 0.5 m. The performance of SAC and MABT in terms of MAD (mean absolute deviation), MSE (mean square error), and MAPE (mean absolute percent error) are also demonstrated in Figure 13. This paper discusses the impact of the highest recognition rate within the time limit when distances between tags are the same and fixed, and when they are not the same, but maintain an average distance. Figure 13a,b shows one row of labels on the conveyor with a label density of 2. Figure 13c,d shows two rows of labels on the conveyor, with a label density of 4. As the results of Figure 13, the MAD, MSE, and MAPE values of MABT are significantly lower than those of SAC. This means the successful identification rate of MABT is significantly higher than SAC.

Specifically, Figure 13a shows that, as the rate of the conveyor belt sped up, so did the number of tags entering the coverage area of the reader per second, so that the tag recognition rate gradually declined. The recognition rate of MABT began to decrease after the speed reached 13 m/s, and the recognition rate of SAC began to decrease continuously after the speed reached 10 m/s. This is because, in the SAC algorithm, when the number of labels in the coverage area was larger, the fixed frame length was insufficient to cope with a circumstance in which the amount of labels is larger than the frame length. On the other hand, when there was no estimated frame, it was easy to underestimate the number of tags, which led to an excessive amount of collisions during the identification process. In comparison, MABT performed excellently at higher speeds. As shown in Figure 13b, the distance between tags was averaged, but the distance between each tag was different. At a speed of 11 m/s, the SAC recognition rate was only 39.95%, while the MABT recognitions rate was still 97.60%, a difference of nearly 60%. There are two reasons for this: (1) The MABT algorithm used a more accurate estimation of optimal frame size so that the frame could quickly adjust the number of processes required, greatly reducing the incidence of underestimated labels. (2) When distance was exponentially assigned, it meant that the number of tags entered per second was also different, but an average value was maintained. In addition to avoiding excessive collisions, the use of dynamic frames also reduced unnecessary time slot waste. With as rates increased, the difference in recognition rate between the two narrowed. However, under the requirement of maximum movement rate and better the recognition rate, MABT significantly improved on SAC (Figure 13b). To maintain a recognition rate of more than 90%, SAC could only be about 9.9 m/s at the most, while MABT could reach about 11.4 m/s, which is equivalent to processing 22.8 labels per second. When the density of tags on the conveyor belt was further increased, as shown in Figure 13c,d, it can be seen that, when the distance between tags was fixed, the difference between the two recognition rates was about 30%. The difference in recognition rates of the two was about 10% when the distance between tags was not uniform. Therefore, Figure 13 shows that, in the context of dynamic labels on a conveyor belt, to achieve the highest recognition rate in a limited time, the MABT algorithm provides high recognition performance at high rates of speed.

**Figure 14:**
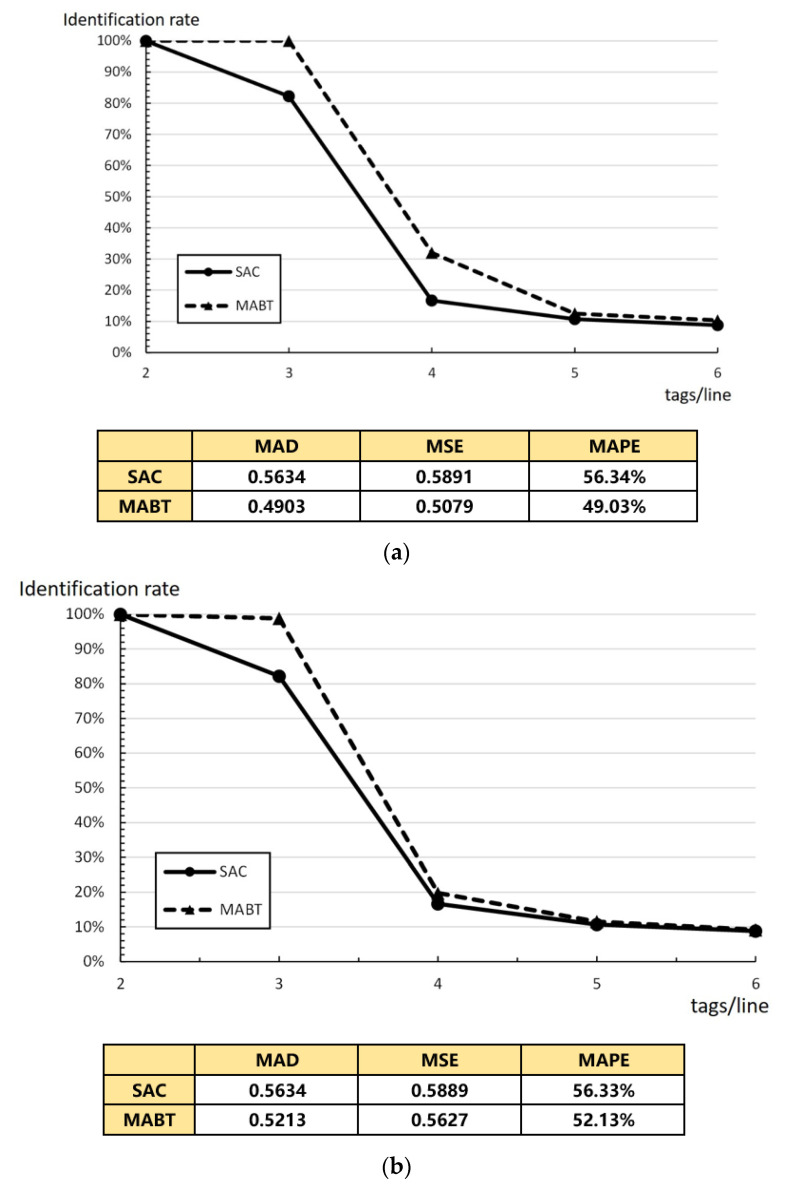
Average tag number per column: (**a**) fixed *n* tags/line; and (**b**) average *n* tags/line.

Figure 14 shows that, at a fixed rate and fixed intervals, the identification rate changes when the average number of tags per line changes from 2 to 5. For example, suppose the moving speed of the conveyor is 4 m/s and there are a large number of tags within the time limit, i.e., the impact on the recognition rate when the number of tags on each line is the same and fixed and when the number of tags is changed.

Figure 14 also demonstrates that the MAD, MSE, and MAPE values of MABT are lower than that of SAC. This means the successful identification rate of MABT is higher than SAC even when the tag arrival rate is fixed. As shown in Figure 14a,b, the average number of labels on each line of the conveyor is 2 and average label density is 4. The average number of tags entering coverage per second is 16. The average number of labels on each line of the conveyor is 3 and the average label density is 6. The average number of tags entering coverage per second is 24. The difference is that, in Figure 14a, there is a fixed number in each row, while, in Figure 14b, the number in each row is different but the total average is the same as that of Figure 14a. In the pursuit of maximum density combined with an acceptable recognition rate, MABT is a significant improvement on SAC. The reason can be seen in Figure 13, which shows that, when density on the conveyor belt increases, it becomes more difficult for the reader to identify the tags. If the fixed frame length of SAC is adopted, collisions are more likely to occur. MABT adjusts the optimal frame size more quickly by more accurately estimating collisions and the expected increase in the number of tags. Although the difference in the recognition rate between two algorithm decreases as the speed of the conveyor belt increases, when we compared the difference in recognition rates at three labels per line, SAC comes in at about 82.20%, while MABT is at 98.85% in both Figure 14a,b.

**Figure 15:**
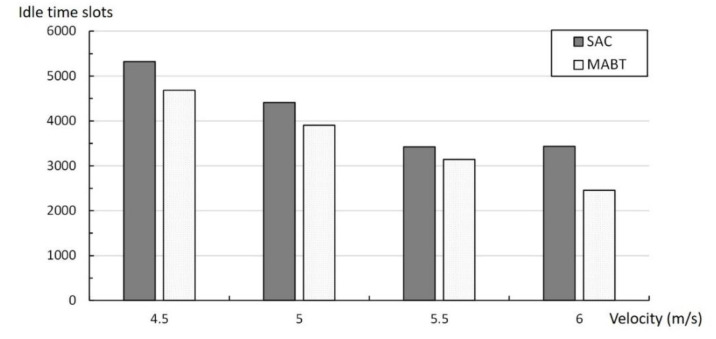
Total number of time slots used at different rates.

**Figure 16:**
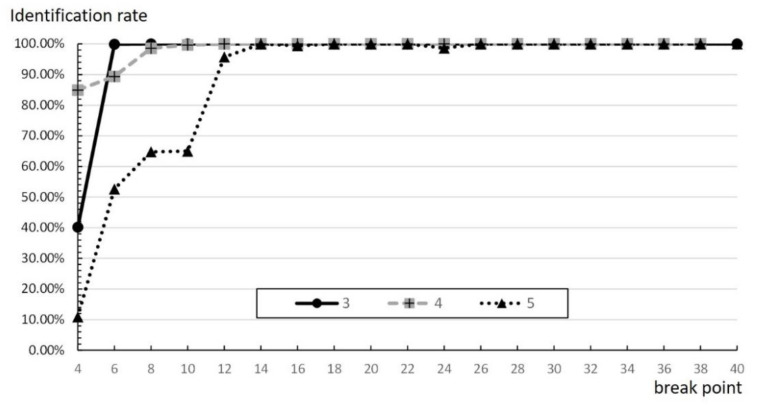
Identification rate of different velocities at different breakpoints.

The purpose of setting a break point is to ensure that the limited time slots can be properly used to reduce the number of idle time slots and collision time slots, thereby increasing the probability of successful tag identification. Therefore, Figure 15 and Figure 16 show the effect of the break point on the usage of the time slots and the label groups. Figure 15 shows the total idle time slots for SAC and MABT during the identification process. The figure shows that, from 4.5 to 6 m/s, the total number of idle time slots required by MABT as a whole is about 19% less than SAC. There are two possible reasons for the small difference between the speeds of 4.5 and 5.5 m/s. One is that the recognition rate of both algorithms is nearly 90% or more and the other is either that the recognition rate of the reader is greater than the moving speed of the conveyor belt or that the interval between the tags is greater than the coverage of the reader. It is impossible to avoid the occurrence of a slot when idle.

In addition, Figure 16 shows that improper break points may also cause a low recognition rate. When the number of time slots at the break point is 14 or fewer, no matter what the rate is, the slower is the occurrence of the break point, the higher is the recognition rate, which reduces influence between groups. However, when the number of time slots at the break point is more than 14 time slots, there is no significance. A possible reason for this is that the value of the break point is too large. It also means that the break point is larger than the average number of the actual time slots, thus it does not affect the number of groups or time slots at all.

## 5. Conclusions

We propose the MABT algorithm to deal with the problem of dynamic tag identification performance, and to achieve an acceptable tag identification rate at high tag densities and high tag movement speeds. The MABT responds more accurately to tags in a rapidly changing conveyor belt transmission environment. It can estimate the next appropriate frame length from current tag identification status, which leads to effective use of idle time slots and reduced collisions. It also achieves better workload optimization and identification time prioritization. Simulation results indicate that MABT recognition rate increases by about 20% over SAC when the rate of movement is 6 m/s and average label density is 4 per meter. Additionally, at a rate of movement of 4 m/s while recognition rate is kept at 98%, the average label density per meter increases by about 17%. Therefore, in addition to solving problems with traditional algorithms, MABT also has significant advantages over existing SAC algorithms. The proposed MABT algorithm can be implemented by using Request Type B (REQB) command and Slot Marker Command in the RFID firmware based on the chosen commercial RFID system [25,26].

The two factors in the dynamic tag model combine to make four kinds of dynamic tags: a single fixed quantity, a single variable quantity, a dynamic fixed quantity, and a dynamic variable quantity. At present, MABT can only be considered a solution for single fixed quantities and dynamic fixed quantities. Therefore, in the future, we will further adjust the MABT algorithm to make it better integrate into different applications and scenarios [27,28,29,30].

## Figures and Tables

**Table 1:** Definition of related parameters.

Parameter	Definition
*L*	Coverage area of identity reader
*d*	Average distance between adjacent tags
*p*	Average density of tags per meter on the conveyor belt
*f*	Frame length
*s, s.id, cumSlot*	time slot, time slot code, accumulated number of time slots
*Ni, Ns, Nc* *Fi, Fs, Fc*	Total number of idle slots, success slots, and collision slots Number of idle, success, and collided states of the original frame
*frameId*	ID of frame
*curGroupId*	ID of currently identified group
*maxGroupId*	Maximum allowable number of group IDs
*groupLen*	Total number of groups on the conveyor belt
*groupNum*	Total number of groups within the coverage of the reader
*groupSize*	Average number of tags in group
*Finish* [*x*]	Number of identified tags in group x
*breakPoint*	Restricted identification time under each super frame
*RC*	Reader time slot counter
*TC*	Time slot counter for each tag

**Table 2:** Definition of parameters in the simulated environment.

Parameter	Definition
*r*	Identification rate
*L*	Identification reader coverage
*d*	Average distance between adjacent tags
*v*	Moving speed of tag on the conveyor belt
*column*	Average number of tags per line on the conveyor belt
*p*	Average tag density per meter on the conveyor belt
*T_0_*	Duration of time slot
*breakPoint*	Identification deadline in each frame
